# Integrated Meta-omics Reveals a Fungus-Associated Bacteriome and Distinct Functional Pathways in Clostridioides difficile Infection

**DOI:** 10.1128/mSphere.00454-19

**Published:** 2019-08-28

**Authors:** David B. Stewart, Justin R. Wright, Maria Fowler, Christopher J. McLimans, Vasily Tokarev, Isabella Amaniera, Owen Baker, Hoi-Tong Wong, Jeff Brabec, Rebecca Drucker, Regina Lamendella

**Affiliations:** a Department of Surgery, University of Arizona, Tucson, Arizona, USA; b Department of Biology, Juniata College, Huntingdon, Pennsylvania, USA; c Contamination Source Identification, LLC, Huntingdon, Pennsylvania, USA; Arizona State University

**Keywords:** *Clostridioides difficile*, metagenomics, metatranscriptomics, microbiome, mycobiome

## Abstract

There has been no prior application of matched metagenomics and metatranscriptomics in Clostridioides difficile infection (CDI) evaluating the role of fungi in CDI or identifying community functions that contribute to the development of this disease. We collected diarrheal stools from 49 inpatients (18 of whom tested positive for CDI) under stringent inclusion criteria. We utilized a tiered sequencing approach to identify enriched bacterial and fungal taxa, using 16S and internal transcribed spacer (ITS) rRNA gene amplicon sequencing, with matched metagenomics and metatranscriptomics performed on a subset of the population. Distinct bacterial and fungal compositions distinguished CDI-positive and -negative patients, with the greatest differentiation between the cohorts observed based on bacterial metatranscriptomics. Bipartite network analyses demonstrated that *Aspergillus* and *Penicillium* taxa shared a strong positive relationship in CDI patients and together formed negative cooccurring relationships with several bacterial taxa, including the *Oscillospira*, *Comamonadaceae*, *Microbacteriaceae*, and *Cytophagaceae*. Metatranscriptomics revealed enriched pathways in CDI patients associated with biofilm production primarily driven by Escherichia coli and *Pseudomonas*, quorum-sensing proteins, and two-component systems related to functions such as osmotic regulation, linoleic acid metabolism, and flagellar assembly. Differential expression of functional pathways unveiled a mechanism by which the causal dysbiosis of CDI may self-perpetuate, potentially contributing to treatment failures. We propose that CDI has a distinct fungus-associated bacteriome, and this first description of metatranscriptomics in human subjects with CDI demonstrates that inflammation, osmotic changes, and biofilm production are key elements of CDI pathophysiology.

**IMPORTANCE** Our data suggest a potential role for fungi in the most common nosocomial bacterial infection in the United States, introducing the concept of a transkingdom interaction between bacteria and fungi in this disease. We also provide the first direct measure of microbial community function in Clostridioides difficile infection using patient-derived tissue samples, revealing antibiotic-independent mechanisms by which C. difficile infection may resist a return to a healthy gut microbiome.

## Abstract (precis)

Our data suggest a potential role for fungi in the most common nosocomial bacterial infection in the United States, introducing the concept of a transkingdom interaction between bacteria and fungi in this disease. We also provide the first direct measure of microbial community function in Clostridioides difficile infection using patient-derived tissue samples, revealing antibiotic-independent mechanisms by which C. difficile infection may resist a return to a healthy gut microbiome.

## INTRODUCTION

Clostridioides difficile infection (CDI), the most common etiology of nosocomial infectious diarrhea, is caused by an intestinal dysbiosis that is virtually always attributable to antibiotics. There is a growing body of data showing that distinct bacterial and fungal community structures distinguish the dysbiotic state of CDI from antibiotic-associated diarrhea due to other causes (1–4). Our group has previously observed (5) that CDI is associated with the enrichment of microbial taxa with both mucinolytic activity as well as with the capacity to produce xenobiotic compounds with possible antibiotic-like effects, all of which may perpetuate the dysbiotic state and, thus, CDI itself. Based on work focused on cooccurrence network modeling, our group has also observed (5) a possible transkingdom interaction in CDI between fungal and bacterial consortia, while CDI-negative control patients with diarrhea demonstrated virtually no fungal enrichment. Additionally, predictive metagenomics tools revealed the enrichment of pathways in CDI associated with lipopolysaccharide and arachidonic acid synthesis as potential proinflammatory mediators, as well as revealing enrichment of pathways associated with glycan and xenobiotic biosynthesis, further explaining why C. difficile is selectively advantaged to exploit new environmental niches created through compositional and functional perturbations introduced in dysbiotic states.

Previous studies on CDI have generally been restricted to the amplification of small-subunit RNA (16S rRNA gene) to identify the bacterial communities enriched in CDI. Utilization of internal transcribed spacer (ITS) rRNA sequencing as a target for fungal community profiling is usually omitted from these studies, leading to a paucity of data regarding the role of the mycobiome in CDI (6). In addition, to date there has been limited application of metagenomics (MG) and no application of metatranscriptomics (MT) to the study of CDI in humans, in part due to their high cost. MG allows for the sequencing of genes beyond 16S rRNA, enabling a description of the underlying genetic potential of a given microbial community, while MT analysis reveals functionally active bacteria within an ecosystem. When combined, these techniques identify the organisms, and their activities, most important to a disease state (7).

In this study, we provide the first published merged omics data comparing inpatients with diarrhea, with and without CDI, by using whole-metagenome shotgun sequencing and metatranscriptomics. We discuss several novel functional pathways by which the CDI disease state may persist, independent of issues of antibiotic resistance. Additionally, we examine a role for fungal organisms in CDI.

## RESULTS

### Description of study population.

The mean age of CDI patients was 65.3 ± 17 years, while non-CDI subjects had a mean age of 60 ± 18 years (*P = *0.32 by *t* test). There was no difference in gender or the mean number of chronic comorbidities harbored by patients in either cohort (*P > *0.05), with the most common chronic medical conditions being systemic hypertension, non-insulin-dependent diabetes mellitus, and coronary artery disease. There was also no difference in the incidence of antibiotic use prior to stool samples being collected, either in terms of presence or absence of antibiotics or in terms of number of antibiotics (*P > *0.05). Metadata can be found in Fig. S1 in the supplemental material. Bacterial and fungal community profiling is described in the supplemental material (Text S2).

10.1128/mSphere.00454-19.2 FIG S1 LEfSe cladograms display significantly enriched (LDA > 3.0; *P < *0.05) bacterial (left) and fungal (right) taxa within CDI^+^ (red) and CDI^−^ (green) sample groups. Download FIG S1, TIF file, 0.3 MB. Copyright © 2019 Stewart et al. 2019 Stewart et al. This content is distributed under the terms of the Creative Commons Attribution 4.0 International license.

### Insignificant differences in fungal and bacterial species richness and evenness observed between CDI^+^ and CDI^−^ cohorts.

Considering the 16S data set, bacterial community species richness and evenness was not found to be significantly different between CDI^+^ and CDI^−^ cohorts (observed richness, *P = *0.076; Heip’s evenness, *P = *0.841) despite an observed lower average species richness within CDI^+^ samples (CDI^+^ observed species mean, 93.408 ± 23.12; CDI^−^ observed species mean, 121.76 ± 58.26). A significant decrease in alpha diversity within CDI^+^ samples was observed using the PD whole-tree metric (*P = *0.015). Fungal community species richness and evenness were not significantly different between CDI^+^ and CDI^−^ cohorts (observed richness, *P = *0.26; Heip’s evenness, *P = *0.112). A summary of alpha diversity comparison statistics can be found in Table 1.

**TABLE 1:** 16S and ITS alpha diversity measures within CDI^+^/CDI^−^ individuals

Alpha diversity measure	CDI^+^ mean	CDI^−^ mean	*t* statistic	*P* value
16S rRNA				
Observed	93.41 (±23.12)	121.77 (±58.26)	−1.90	0.076
PD whole tree	9.08 (±2.24)	11.99 (±4.88)	−2.34	0.015
Heip's evenness	0.079 (±0.035)	0.0769 (±0.042)	0.21	0.84
ITS				
Observed	57.76 (±23.37)	65.85 (±17.82)	1.14	0.26
Heip's evenness	0.092 (±0.10)	0.047 (±0.057)	−1.67	0.112

### Significantly different bacterial and fungal community structures exist within CDI^+^ and CDI^−^ individuals.

**FIG 1:**
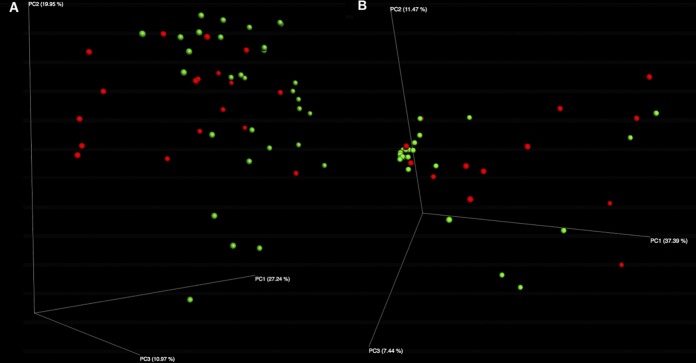
Principal coordinate analysis (PCoA) plots reveal significantly differential bacterial (A) and fungal (B) community compositions between CDI^+^ and CDI^−^ cohorts. PCoA plots present significant clustering of CDI^+^ and CDI^−^ samples based on UniFrac distances in bacterial (A) and weighted Jaccard distances for fungal (B) community composition (bacterial ANOSIM, *P = *0.022; fungal ANOSIM, *P = *0.038).

Principal coordinate analysis (PCoA) plots revealed significant differences in bacterial and fungal community composition between CDI^+^ and CDI^−^ cohorts. This is demonstrated by distinct clustering between the disease states (Fig. 1A and B) and was significant considering both 16S and ITS data sets (16S analysis of similarity [ANOSIM], *P = *0.022; ITS ANOSIM, *P = *0.038). Multivariate association with linear model (MaAsLin) analysis of bacterial abundance data is summarized in Table 2 and revealed a total of seven significantly enriched bacterial biomarkers and no significantly enriched fungal biomarker within the CDI^−^ cohort (Tables 2 and 3). Within the CDI^+^ cohort, nine bacterial and two fungal significantly enriched biomarker taxa were identified. Bacterial taxa, including *Faecalibacterium* and *Collinsella*, were enriched within the CDI^−^ cohort. Bacterial taxa within the *Clostridiaceae*, *Peptostreptoccocaceae*, and *Enterococcus* were identified as significantly enriched biomarkers within the CDI^+^ cohort. We identified the fungal genera *Byssochlamys* and *Helotiales* as significantly enriched within the CDI^+^ cohort.

**TABLE 2:** 16S rRNA MaAsLin enrichment results with relative abundance quartiles

Taxon	Enrichment (%) for^*a*^:				Coefficient	Enrichment group	*P* value	FDR-corrected *P* value
	CDI^−^ Q1	CDI^−^ Q3	CDI^+^ Q1	CDI^+^ Q3				
*Flavobacterium*	0.0250	0.0455	0.0000	0.0105	0.01	CDI^−^	0.000	0.000
*Comamonadaceae* spp.	0.0168	0.0301	0.0009	0.0112	0.01	CDI^−^	0.000	0.000
*Stramenopiles* spp.	0.0095	0.0152	0.0003	0.0044	0.01	CDI^−^	0.000	0.000
*Collinsella*	0.0000	0.1491	0.0000	0.0000	0.00	CDI^−^	0.000	0.002
*Coriobacteriaceae* spp.	0.0000	0.0430	0.0000	0.0007	0.00	CDI^−^	0.017	0.206
*Barnesiellaceae* spp.	0.0000	0.0113	0.0000	0.0000	0.00	CDI^−^	0.046	0.422
*Faecalibacterium*	0.0000	0.1751	0.0000	0.0000	0.03	CDI^−^	0.048	0.422
*Proteus*	0.0000	0.0000	0.0127	0.0445	−0.01	CDI^+^	0.000	0.000
*Synechococcus*	0.0000	0.0000	0.0016	0.0322	−0.01	CDI^+^	0.000	0.000
*Turicibacter*	0.0000	0.0000	0.0111	0.0288	−0.01	CDI^+^	0.000	0.000
*Peptostreptococcaceae* spp.	0.0000	0.0179	0.0846	2.5594	−0.07	CDI^+^	0.000	0.000
*Bacteroidales* sp. strain S24-7	0.0000	0.0000	0.0073	0.1120	−0.02	CDI^+^	0.000	0.000
*Clostridiaceae* spp.	0.0000	0.0668	0.0350	0.3970	−0.02	CDI^+^	0.002	0.030
*Clostridium*	0.0000	0.0125	0.0012	0.0182	0.00	CDI^+^	0.012	0.175
*Dorea*	0.0007	0.1724	0.0024	2.1379	−0.03	CDI^+^	0.029	0.336
*Enterococcus*	0.0003	0.3498	0.0137	0.4099	−0.01	CDI^+^	0.034	0.357

a Q1, quartile 1; Q3, quartile 3.

**TABLE 3:** ITS MaAsLin enrichment results with relative abundance quartiles

Taxon	Enrichment (%) for:				Coefficient	Enrichment group	*P* value	FDR-corrected *P* value
	CDI^−^ Q1	CDI^−^ Q3	CDI^+^ Q1	CDI^+^ Q3				
*Byssochlamys*	0.000	0.000	0.000	0.252	−0.10	CDI^+^	0.04	0.402294227
*Helotiales* spp.	0.000	0.000	0.000	0.009	−0.02	CDI^+^	0.05	0.402294227

### Network analysis reveals negative interactions between fungi and commensal, butyrate-producing bacteria within the gut of CDI^+^ individuals.

**FIG 2:**
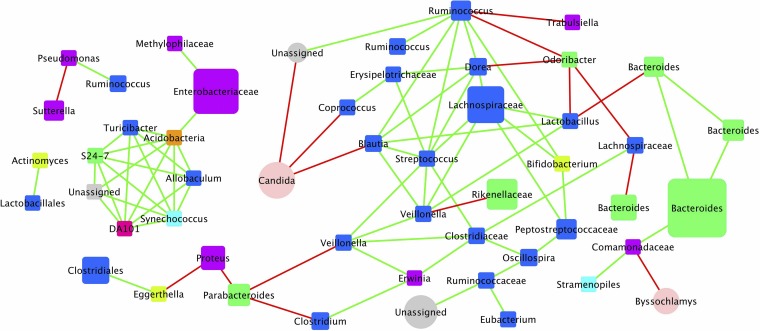
Bipartite cooccurrence network plot of bacterial and fungal taxa within the fecal microbiome of CDI^+^ patients. The cooccurrence network plot generated within the Cytoscape plugin *Conet* revealed strong positive and negative correlations between OTUs summarized at the genus level identified within CDI^+^ stool samples. Each node on the plot is representative of a single bacterial (square) or fungal (circle) taxon, and nodes are colored by phyla. Edges connecting nodes highlighted in green represent strong positive correlations, whereas edges highlighted in red represent strong negative correlations.

Bipartite networks for CDI^+^ individuals displayed negative cooccurring relationships between fungi and commensal gut bacteria (Fig. 2). *Candida* and *Byssochlamys* were present at relatively high abundances within CDI^+^ individuals and formed strong negative relationships with several bacterial taxa, including *Coprococcus*, *Blautia*, and *Comamonadaceae*, as well as an unassigned fungus. The same unassigned fungus also had a strong positive relationship with the *Ruminococcaceae*. No negative cooccurring relationships were observed between fungal and bacterial taxa when considering the CDI^−^ data set (Fig. S2). A single positive interaction between an unassigned fungus and *Bacteroides* was observed within the CDI^−^ cohort and marks the only transkingdom relationship within the network. Also, there is a positive interaction between two *Candida* species that do not have any relationship with any of the commensal gut bacteria.

10.1128/mSphere.00454-19.3 FIG S2 Bipartite cooccurrence network plot of bacterial and fungal taxa within the fecal microbiome of CDI^−^ patients. Each node on the plot is representative of a single bacterial (square) or fungal (circle) taxon. Nodes are colored by respective phylum. Edges connecting nodes highlighted in green denote strong positive correlations, whereas edges highlighted in red denote strong negative correlations. Here, no negative interactions between fungal and bacterial taxa can be identified within the CDI^−^ network. Download FIG S2, TIF file, 0.3 MB. Copyright © 2019 Stewart et al. 2019 Stewart et al. This content is distributed under the terms of the Creative Commons Attribution 4.0 International license.

### Metatranscriptomic data reveal greater differentiation between CDI^+^ and CDI^−^ individuals than did metagenomic comparisons.

**FIG 3:**
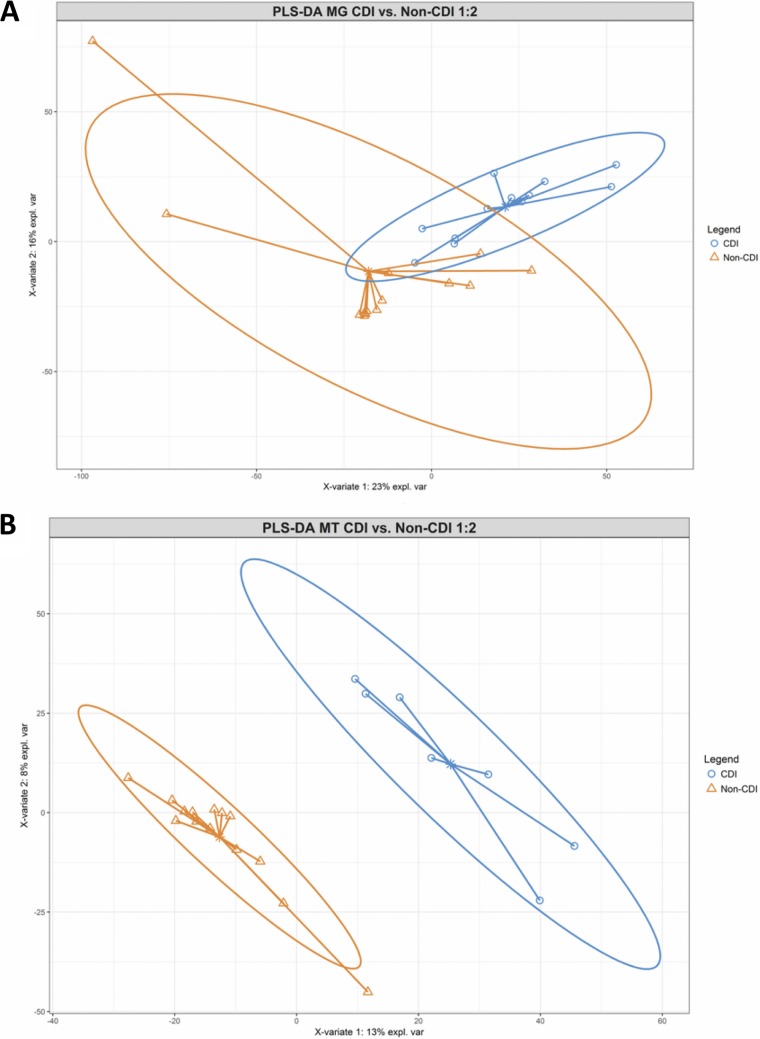
Partial least-squares discriminant analysis (PLS-DA) score plot of metagenomic (A) and metatranscriptomic (B) gene abundance data sets of CDI^+^ and CDI^−^ individuals. Two-dimensional PLS-DA plots generated within MixOmics reveal distinct clustering of CDI^+^ (blue) and CDI^−^ (orange) samples using CPM-normalized counts of metagenome functional gene data and metatranscriptome expression data, indicating distinct functional gene and expression profiles between disease states. Complementary area under the receiver operating characteristic (AUROC) measures were calculated to validate the metagenome (AUROC of 0.934) and metatranscriptome (AUROC of 1.0) PLS-DA models. An improved ability for the model to differentiate between CDI^+^ and CDI^−^ samples was observed for the metatranscriptome data set.

To visualize overall differences in metagenome and metatranscriptome functional gene profiles between disease states, partial least-squares discriminant analysis (PLS-DA) was conducted. Counts per million (CPM)-normalized reads per kilobase KEGG orthology (KO) counts from respective metagenome and metatranscriptome data sets were used to create a PLS-DA model comparing CDI status, which resulted in clustering between disease states for both data sets (Fig. 3A and B). Greater differentiation between CDI^+^ and CDI^−^ cohorts was observed within the metatranscriptome PLS-DA model than in the model generated based on metagenome data. The metatranscriptomic PLS-DA model yielded a superior area under the receiver operating characteristic curve (AUROC) measure (AUROC of 1.0) than the metagenomic data set (AUROC of 0.93), indicating an increased ability for the model to differentiate between CDI^+^ and CDI^−^ states when considering metatranscriptome expression data compared to metagenomic gene abundance information.

### Biomarker analysis reveals increased expression of biofilm formation and quorum-sensing gene pathways within CDI^+^ individuals.

Linear discriminant analysis effect size (LEfSe) analysis of metagenomic abundance data and metatranscriptomic expression data revealed bacterial and functional gene pathway biomarkers within CDI^+^ and CDI^−^ individuals. Considering the metagenomic data set, ten significantly enriched (linear discriminant analysis [LDA] > 2.0; *P < *0.05) biomarker taxa were identified within the CDI^+^ cohort, including Clostridioides difficile, Escherichia coli, an unclassified *Peptostreptococcaceae* member, and the *Enterobacteriaceae* (Fig. S3). *Akkermansia munciniphila*, Faecalibacterium prausnitzii, *Coprococcus*, Alistipes shahii, *Collinsella*, and the *Verrucomicrobiaceae* were significantly enriched within the CDI^−^ cohort. Comparable results between the 16S comparison and increased taxonomic resolution were observed in the metagenomic LEfSe analysis between CDI^+^ and CDI^−^ individuals. For example, within the 16S data set, we classified the enrichment of Clostridioides difficile with confidence at the *Peptostreptococacceae* taxonomic rank, whereas within the metagenomic data set we are able to obtain species-level classification of Clostridioides difficile enrichment. The *Faecalibacterium* result was confirmed by shotgun metagenomics as Faecalibacterium prausnitzii and is enriched within the CDI^−^ cohort for both 16S and metagenomic data sets. The metatranscriptome data set yielded 10 and six active biomarker taxa within the CDI^+^ and CDI^−^ cohorts, respectively (Fig. S4). Escherichia coli, Clostridium clostridioforme, and the *Enterobacteriaceae* were among the enriched active bacteria within the CDI^+^ cohort (LDA > 2.0; *P < *0.05). Within the CDI^−^ cohort, the only active taxa identified as significantly enriched belonged to the *Verrucomicrobia* phylum.

10.1128/mSphere.00454-19.4 FIG S3 LEfSe cladograms were generated to display significantly enriched (LDA > 2.0; *P < *0.05) bacterial taxa within CDI^+^ (red) and CDI^−^ (green) samples considering the metagenomic dataset based on the Metaphlan2 taxonomic profiling tool. Taxa including Clostridium difficile, *Enterobacteriaceae*, and Escherichia coli were observed to be significantly enriched within the CDI^+^ cohort. Download FIG S3, TIF file, 0.3 MB. Copyright © 2019 Stewart et al. 2019 Stewart et al. This content is distributed under the terms of the Creative Commons Attribution 4.0 International license.

10.1128/mSphere.00454-19.5 FIG S4 LEfSe cladograms were generated to display significantly enriched (LDA > 2.0; *P < *0.05) bacterial taxa within CDI^+^ (red) and CDI^−^ (green) samples considering the metatranscriptomic dataset based on the Metaphlan2 taxonomic profiling tool. Taxa within the *Clostridium* and *Enterobacteriaceae* were observed to be significantly enriched within the CDI^+^ cohort, whereas taxonomy within the *Verrucomicrobiaceae* was identified as enriched within the CDI^−^ cohort. Download FIG S4, TIF file, 0.3 MB. Copyright © 2019 Stewart et al. 2019 Stewart et al. This content is distributed under the terms of the Creative Commons Attribution 4.0 International license.

To define functional pathways driving shifts in metagenome profiles and metatranscriptome expression data between CDI^+^ and CDI^−^ cohorts, MaAsLin analysis was conducted on summarized KEGG expression data to identify associations between enriched bacterial and functional gene pathways and collected patient metadata. While considering the metagenome data set, 31 significantly enriched summarized functional KEGG pathways were identified within CDI^+^ and CDI^−^ cohorts (Table 4 and Fig. S5). Phosphotransferase systems (2.16-fold increase in CDI^+^ individuals), two-component systems (1.43-fold increase in CDI^+^ individuals), flagellar assembly (2.52-fold increase in CDI^+^ individuals), ABC transporters, and bacterial chemotaxis were the top five level 3 gene pathway biomarkers of CDI^+^ status within the metagenome data set. N-glycan biosynthesis, carbon fixation pathways in prokaryotes, aminoacyl tRNA biosynthesis (1.28-fold increase in CDI^+^ individuals), citrate cycle-tricarboxylic acid (TCA) cycle, and protein processing in endoplasmic reticulum were observed as the top five biomarker pathways of CDI^−^ individuals.

**TABLE 4:** Metagenomic MaAsLin interquartile ranges

Pathway	Enrichment (%) for:				Coefficient	Enrichment group	*P* value	*q* value
	CDI^−^ Q1	CDI^−^ Q3	CDI^+^ Q1	CDI^+^ Q3				
Aminoacyl tRNA biosynthesis	1.48	1.72	1.13	1.47	−0.013	CDI^−^	0.005	0.247
Carbon fixation pathways in prokaryotes	1.78	2.40	1.30	2.07	−0.014	CDI^−^	0.006	0.420
Citrate cycle TCA cycle	1.41	1.88	1.00	1.56	−0.013	CDI^−^	0.009	0.420
Novobiocin biosynthesis	0.21	0.34	0.17	0.25	−0.009	CDI^−^	0.013	0.288
Prodigiosin biosynthesis	0.18	0.25	0.13	0.17	−0.007	CDI^−^	0.028	0.229
Protein processing in endoplasmic reticulum	0.06	0.12	0.01	0.06	−0.012	CDI^−^	0.034	0.229
Terpenoid backbone biosynthesis	0.63	0.91	0.54	0.69	−0.007	CDI^−^	0.041	0.465
Valine leucine and isoleucine biosynthesis	0.97	1.10	0.76	0.94	−0.007	CDI^−^	0.042	0.420
Various types of N-glycan biosynthesis	0.06	0.24	0.00	0.11	−0.017	CDI^−^	0.050	0.367
ABC transporters	2.31	3.83	3.35	6.18	0.037	CDI^+^	0.003	0.420
Alpha-linolenic acid metabolism	0.00	0.03	0.04	0.13	0.014	CDI^+^	0.004	0.229
Ascorbate and aldarate metabolism	0.10	0.28	0.22	0.35	0.015	CDI^+^	0.005	0.342
Bacterial chemotaxis	0.03	0.11	0.18	0.40	0.025	CDI^+^	0.005	0.229
Biofilm formation, Pseudomonas aeruginosa	0.06	0.17	0.17	0.40	0.019	CDI^+^	0.005	0.229
Biofilm formation, Vibrio cholerae	0.31	0.58	0.64	0.96	0.018	CDI^+^	0.007	0.316
Chlorocyclohexane and chlorobenzene degradation	0.00	0.01	0.01	0.03	0.009	CDI^+^	0.007	0.229
Ether lipid metabolism	0.00	0.02	0.01	0.04	0.007	CDI^+^	0.007	0.342
Ethylbenzene degradation	0.00	0.02	0.02	0.09	0.014	CDI^+^	0.013	0.229
Flagellar assembly	0.00	0.06	0.18	0.81	0.038	CDI^+^	0.014	0.229
Fluorobenzoate degradation	0.00	0.01	0.01	0.02	0.007	CDI^+^	0.015	0.420
Geraniol degradation	0.00	0.02	0.02	0.09	0.014	CDI^+^	0.017	0.229
Glutathione metabolism	0.30	0.46	0.50	0.62	0.011	CDI^+^	0.020	0.362
Glycerolipid metabolism	0.24	0.35	0.29	0.60	0.009	CDI^+^	0.021	0.420
Linoleic acid metabolism	0.00	0.01	0.01	0.04	0.007	CDI^+^	0.022	0.301
Naphthalene degradation	0.00	0.06	0.03	0.07	0.009	CDI^+^	0.026	0.420
Phosphotransferase system	0.11	0.77	0.76	1.95	0.047	CDI^+^	0.028	0.229
Propanoate metabolism	0.83	1.00	1.01	1.20	0.008	CDI^+^	0.036	0.367
Retinol metabolism	0.00	0.04	0.02	0.05	0.007	CDI^+^	0.039	0.420
Sulfur metabolism	0.47	0.82	0.80	1.23	0.018	CDI^+^	0.040	0.288
Toluene degradation	0.00	0.01	0.01	0.02	0.007	CDI^+^	0.040	0.342
Two-component system	1.15	1.95	1.95	3.12	0.040	CDI^+^	0.043	0.288

Within the metatranscriptome data set, we identified 29 enriched KEGG pathways (Table 5). ABC transporters (1.44-fold increase in CDI^+^ individuals), two-component system (1.85-fold increase in CDI^+^ individuals), flagellar assembly (5.11-fold increase in CDI^+^ individuals), phosphotransferase system (1.57-fold increase in CDI^+^ individuals), and ascorbate and adorate metabolism (3,47-fold increase in CDI^+^ individuals) were the top five expressed gene pathways identified within the CDI^+^ cohort. Additional enriched pathways, including quorum sensing (1.19-fold increase in CDI^+^ individuals), bacterial chemotaxis (3.66-fold increase in CDI^+^ individuals), linoleic acid metabolism (12.19-fold increase in CDI^+^ individuals), and biofilm formation-Pseudomonas aeruginosa (3.31-fold increase in CDI^+^ individuals), were also observed within the CDI^+^ expression data. Protein processing in the endoplasmic reticulum, valine, leucine, and isoleucine biosynthesis, histidine metabolism, one-carbon pool by folate, and carbon fixation in photosynthetic organisms (1.55-fold increase in CDI^−^ individuals) were the top five expressed level 3 KEGG pathways identified as enriched within the CDI^−^ cohort.

**TABLE 5:** Metatranscriptomic MaAsLin enrichment results with relative abundance quartiles

Pathway	Enrichment (CPM) for:				Coefficient	Enrichment group	*P* value	FDR-corrected *P* value
	CDI^−^ Q1	CDI^−^ Q3	CDI^+^ Q1	CDI^+^ Q3				
Valine leucine and isoleucine biosynthesis	9,514.11	15,392.90	5,648.81	8,337.13	−0.02054	CDI^−^	0.008	0.146
Carbon fixation in photosynthetic organisms	10,333.69	14,029.93	6,935.82	8,324.76	−0.01236	CDI^−^	0.008	0.146
Protein processing in endoplasmic reticulum	141.98	2,011.25	0.00	35.63	−0.0226	CDI^−^	0.024	0.255
Glycine serine and threonine metabolism	17,065.75	24,255.21	13,585.16	17,256.03	−0.0103	CDI^−^	0.025	0.255
Histidine metabolism	8,980.54	13,015.11	6,643.91	8,720.96	−0.01492	CDI^−^	0.032	0.301
One-carbon pool by folate	8,894.96	14,537.30	6,096.51	8,641.10	−0.01285	CDI^−^	0.056	0.390
C branched dibasic acid metabolism	5,504.79	9,086.76	4,008.12	6,235.85	−0.01223	CDI^−^	0.059	0.392
Alpha-linolenic acid metabolism	0.00	207.16	693.91	1,265.72	0.02426	CDI^+^	0.000	0.029
Bacterial chemotaxis	167.31	1,297.16	2,265.06	4,147.41	0.03489	CDI^+^	0.000	0.029
Ascorbate and aldarate metabolism	526.98	2,203.76	3,161.59	6,267.15	0.03851	CDI^+^	0.000	0.029
Ethylbenzene degradation	0.00	180.42	497.38	1,033.91	0.02145	CDI^+^	0.001	0.029
Geraniol degradation	0.00	511.95	848.86	1,080.13	0.02247	CDI^+^	0.001	0.029
Flagellar assembly	0.00	1,133.64	1,080.82	7,448.64	0.04871	CDI^+^	0.001	0.029
Two-component system	14,066.55	21,493.11	25,306.60	40,448.82	0.06355	CDI^+^	0.001	0.029
ABC transporters	28,206.82	44,320.11	48,815.21	59,718.37	0.05873	CDI^+^	0.002	0.090
Sulfur relay system	2,878.55	6,146.67	6,467.43	7,358.35	0.02199	CDI^+^	0.003	0.090
Linoleic acid metabolism	0.00	2.72	20.77	344.47	0.00917	CDI^+^	0.003	0.090
Ether lipid metabolism	0.00	26.61	20.77	344.47	0.00915	CDI^+^	0.003	0.090
Chlorocyclohexane and chlorobenzene degradation	0.00	21.93	125.99	506.71	0.01062	CDI^+^	0.004	0.090
Biosynthesis of siderophore group nonribosomal peptides	37.17	647.97	629.22	2,408.83	0.0236	CDI^+^	0.005	0.120
Fluorobenzoate degradation	0.00	5.39	59.17	392.77	0.01066	CDI^+^	0.009	0.165
Sulfur metabolism	4,620.15	9,094.60	7,747.39	11,277.50	0.02683	CDI^+^	0.010	0.165
Toluene degradation	0.00	33.18	66.83	336.92	0.0094	CDI^+^	0.014	0.206
Quorum sensing	14,686.72	18,688.39	18,603.71	23,100.57	0.02664	CDI^+^	0.023	0.255
Phosphotransferase system	4,091.52	9,124.89	11,965.61	14,977.45	0.04152	CDI^+^	0.029	0.279
Mitogen-activated protein kinase signaling pathway, fly	0.00	410.01	403.97	1,096.53	0.01406	CDI^+^	0.033	0.306
Lysine degradation	527.14	2,350.37	1,856.78	2,798.06	0.0183	CDI^+^	0.042	0.345
Proteasome	0.00	5.99	0.00	228.25	0.00495	CDI^+^	0.054	0.390
Biofilm formation, Pseudomonas aeruginosa	1.33	1,326.79	989.98	1,979.08	0.01642	CDI^+^	0.055	0.390

Both metatranscriptomic and metagenomic analyses yielded similar results regarding identified biomarker taxa within CDI^+^ and CDI^−^ individuals. Taxa within the *Enterobacteriaceae* and *Clostridiaceae* were identified to be enriched within the CDI^+^ group for both data sets, whereas the CDI^−^ group yielded enrichment of the *Verrucomicrobia* taxa for both the metagenome and metatranscriptome. When considering functional KEGG pathways associated with CDI^+^ individuals, 12 pathways are common to both the metatranscriptomic and metagenomic analysis. More specifically, the two-component system and the phosphotransferase system were among the top five gene pathway biomarkers of CDI^+^ status within the metagenome and metatranscriptome data set. Only the metatranscriptome revealed association of elevated quorum-sensing gene expression with CDI^+^ status, as this pathway was not significantly differential considering the metagenome data described alone.

**FIG 4:**
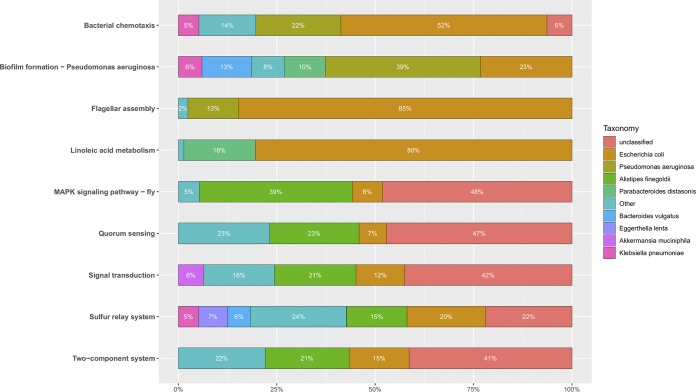
Relative contribution summary was generated to identify taxa contributing to each respective enriched functional gene pathway within the CDI^+^ cohort considering the metatranscriptome data set.

To better understand the taxa contributing to enriched CDI^+^ expression pathways, a taxa contribution plot was generated for selected level 3 enriched KEGG pathways (Fig. 4). The majority of genes found mapped to the bacterial chemotaxis, flagellar assembly, and linoleic acid metabolism pathways within CDI^+^ individuals originated from the E. coli genome (52%, 85%, and 80%, respectively). Pseudomonas aeruginosa was also observed to have distinct contributions to bacterial chemotaxis (22%), biofilm formation (39%), and flagellar assembly pathways (13%). Quorum sensing, signal transduction, sulfur relay system, and two-component systems were predominantly mapped to unclassified bacteria (47%, 42%, 22%, 41%, respectively). Additionally, Alistipes finegoldii and Escherichia coli yielded substantial contributions to quorum-sensing genes within CDI^+^ individuals (23% and 11%, respectively). When specifically visualizing *Clostridioides* expression data of gene pathways within CDI^+^ and CDI^−^ cohorts, an increase in expression of genes related to two-component systems (4.76-fold increase in CDI^+^ individuals) and bacterial secretion systems (2.27-fold increase in CDI^+^ individuals) were observed within CDI^+^ individuals (Fig. S7). Further, an increase in expression of genes related to biofilm formation (2.45-fold increase in CDI^+^ individuals) within the clostridia of CDI^+^ individuals were observed.

**FIG 5:**
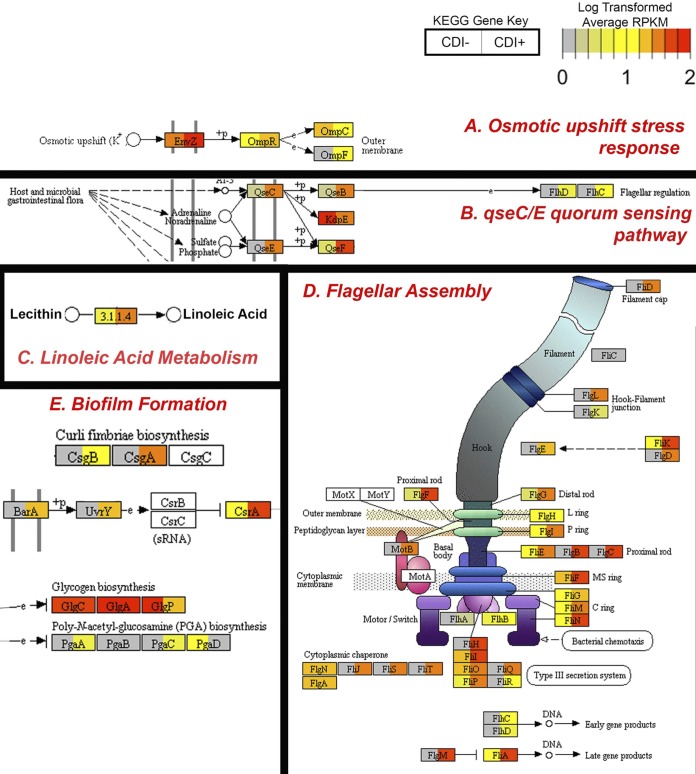
Pathview plots display differences in average CPM-normalized expression counts of KEGG orthologies within CDI^−^ (left) and CDI^+^ (right) cohorts. Average functional expression data were mapped against pathways identified as significantly enriched within CDI^+^ cohorts, including two-component system (A), quorum sensing (B), linoleic acid metabolism (C), flagellar assembly (D), and biofilm formation (E) (LDA > 2.0; *P < *0.05). Each box denotes a functional gene, with the average CDI^−^ cohort expression plotted on the left side of the box and CDI^+^ on the right side. Average relative expression is colored from low expression (gray) to high expression (red). (A) Average elevated expression of genes related to osmotic upshift responses were identified within the CDI^+^ cohort, including the *envZ* osmolarity sensor kinase (3.84-fold increase in CDI^+^ cohort). (B) Increased expression of quorum-sensing *qseC* and *qseE* genes was observed within the CDI^+^ cohort (10.69- and 39.81-fold increase, respectively). (C) Expression of phospholipase *pldA*, involved in the production of linoleic acid, is also elevated within CDI^+^ samples (7.10-fold increase in CDI^+^ cohort). (D) Conserved elevated expression of flagellar assembly genes was observed within CDI^+^ individuals. (E) Elevated expression of genes related to biofilm formation, including curli fimbriae biosynthesis genes *csgB* (40.8-fold increase in CDI^+^ individuals) and *csgA* (52.42-fold increase in CDI^+^ individuals). RPKM, reads per kilobase million.

To visualize differences in average gene expression between CDI^+^ and CDI^−^ cohorts, Pathview plots were generated (Fig. 5A to E). CDI^+^ patients demonstrated enriched two-component systems, linoleic acid metabolism, quorum sensing, flagellar assembly, and biofilm formation pathways. Of note, an increased average expression of two-component osmotic upshift response genes was observed within CDI^+^ individuals; this included increased average expression of the *envZ* osmolarity sensor kinase (3.84-fold increase in CDI^+^ individuals) (Fig. 5A). Within the linoleic acid metabolism pathway, phospholipase *pldA*, involved in the conversion of lecithin to linoleic acid, was observed to be overexpressed within CDI^+^ individuals (7.10-fold increase in CDI^+^ individuals) (Fig. 5B). An increase in average expression of genes within the *qseC* and *qseE* (10.69- and 39.81-fold increase, respectively) quorum-sensing pathway was observed within the CDI^+^ cohort (Fig. 5C). Increased average expression of flagellar assembly genes *fliD*, *flgE*, *flgD*, *flgB*, *flgC*, *fliH*, *fliQ*, *fliR*, *flgM*, *fliJ*, *fliS*, *fliT*, and *motB* was observed within the CDI^+^ cohort compared to levels in CDI^−^ samples (Fig. 5D). Average expression of genes related to biofilm formation was observed to be elevated within the CDI^+^ cohort, including curli fimbriae biosynthesis genes *csgB* (40.8-fold increase in CDI^+^ individuals) and *csgA* (52.42-fold increase in CDI^+^ individuals) (Fig. 5E).

## DISCUSSION

Our study demonstrates that CDI^+^ patients have distinct compositional and functional elements that distinguish them from CDI^−^ subjects, with CDI characterized by a significant enrichment of fungal taxa not observed in C. difficile-negative diarrheal patients. The lack of any difference in preoperative antibiotic use between the cohorts suggests that the enrichment of fungal organisms in CDI is not simply an epiphenomenon caused by antibiotics with fungi coincidentally filling spatial niches left unoccupied by diminished bacterial populations. This finding lends further support to the concept that the dysbiosis associated with CDI has an important contribution from fungal organisms. Direct measurement of gut bacterial community function through metatranscriptomic profiling revealed multiple enriched pathways in CDI involving biofilm formation, bacterial chemotaxis, flagellar assembly, quorum-sensing proteins, xenobiotic production, and various bacterial two-component systems. As discussed below, these data suggest that the dysbiotic state of CDI is associated with community functions that collectively resist a return to a healthy microbial community structure. This information could provide another explanation for the high persistence and recurrence rates of CDI through mechanisms independent of antibiotic resistance. Many of these enriched functional pathways can be traced to E. coli and to *Pseudomonas*, adding to the current literature that describes these common Gram-negative pathogens having an important role in CDI. Our work provides an evaluation of a subject complementary to prior publications regarding bile acid and carbohydrate metabolism in CDI (8–13). We characterize the microbial community structure that characterizes disease-specific features of the dysbiosis in CDI, and in particular, we describe the frequent cooccurrence of several fungal organisms that contribute to that dysbiosis. We also describe the first metatranscriptomic data derived from humans with CDI, providing for the first time several mechanisms that potentially can explain the frequent clinical observation of treatment failures in the medical care of CDI.

The adherence of C. difficile to colonic mucosa contributes both to growth of the organism (14) in the large intestine and intoxication of the host (15). Compositional and functional changes were observed in this study that would contribute to mucosal adherence of C. difficile. The loss of commensal organisms provides a selective advantage to C. difficile by creating spatial niches serving as an early contributor toward C. difficile colonization. Additionally, our data indicate that E. coli and Pseudomonas aeruginosa are majority contributors to pathways dedicated to biofilm formation, suggesting a previously unappreciated sessile microbial biofilm community that may be important in CDI. The role of biofilms in bacterial infections is well known, although the role of biofilms in CDI is poorly studied, including a dearth of information regarding the regulatory mechanisms for biofilm production by C. difficile. The presence of a biofilm may contribute to treatment failures with C. difficile-directed antibiotics; for example, subinhibitory doses of metronidazole have been demonstrated (16) to induce the production of biofilm by C. difficile. Based on multivariate association with linear model (MaAsLin) analysis, biofilm production in this study was simultaneously related both to the use of antibiotics as well as to CDI^+^ status, indicating that antibiotics in general, and CDI in particular, represent two related but distinct environmental cues for biofilm production (Table S1). Whether C. difficile-directed antibiotics also serve as an environmental cue promoting biofilm formation by E. coli and *Pseudomonas* is unknown but warrants further study as a potential contributor to treatment failures.

The enrichment of potentially pathogenic fungal organisms with bacteria known to be associated with human disease states introduces a view of CDI suggesting the potential for a complex transkingdom interaction between bacteria, fungi, and the human host, one mediated in part by quorum sensing. CDI subjects in this study demonstrated significant pathway enrichment for *qseC*, encoding a sensor histidine kinase (17) that serves as part of a bacterial two-component system functioning as a bacterial adrenergic receptor. *qseC* allows for bacterium-host hormonal signaling, with bacteria sensing the production of host norepinephrine as well as allowing for prokaryote-eukaryote signaling through autoinducer-3 (AI-3). In a study focused on enterohemorrhagic Escherichia coli (EHEC) and Citrobacter rodentium, Moreira and colleagues (18) provided the first description of host stress hormones (norepinephrine and epinephrine), which directly affect gut physiology through colonic adrenergic receptors, also having a direct effect on bacterial gene expression, with *qseC* and *qseE* being necessary for C. rodentium successfully colonizing the murine intestine. This only recently appreciated cross talk by which fight-or-flight host hormones signal differential expression of virulence pathways in gut bacteria adds a new dimension to understanding how host stress and inflammatory responses affect the course of gut infections.

These data and recent publications (5, 19) by our group suggest that there is a fungus-associated bacteriome involved in CDI. The consistency over several studies with which a significant enrichment of fungal organisms is observed in human subjects with CDI, but which is not observed in control groups with C. difficile negative diarrhea, suggests that important transkingdom interactions between bacteria and fungi are present in CDI. There is evidence from studies on the rhizosphere (20), on coculture systems (21), and on human subjects with Crohn’s disease (22) indicating that fungi are frequently associated with a particular and environment/disease-specific bacteriome, and that the cooccurrence of these fungal and bacterial organisms is more than a coincidental occupying of the same spatial niche. These prior studies have provided evidence that fungi, when viewed as hosting an associated bacterial community, help to not only protect bacteria from the effects of antibiotics (20) but also potentially allow obligate anaerobic bacteria to grow (21) under aerobic conditions. The mechanisms by which this occurs have not been elucidated, and further, the potential benefits to fungi in this relationship are not clear. In fact, one prior study suggested that *p*-cresol production by C. difficile in culture inhibits hypha formation by C. albicans, which prevents *Candida* from forming a biofilm, suggesting an exploitative relationship between C. difficile and C. albicans.

When combined with what is already known about CDI, the metatranscriptomic data from the present study provides novel insights that may be valuable for understanding how microbial functional pathways contribute to the development of CDI. The use of antibiotics decreases bacterial density and diversity, creating new spatial and metabolic niches that favor the mucosal adherence and population expansion of C. difficile. A potential enhancement to this process is the production of biofilm by C. difficile as well as by E. coli and *Pseudomonas*, which in part may be a response not just to the antibiotics which helped to cause CDI but even those directed at C. difficile itself. At some point thereafter, this is followed by C. difficile intoxication promoted due to multiple factors, including C. difficile reaching a stationary growth phase (23) as well as due to quorum-sensing proteins (24), the latter of which may involve pathways by which host stress hormones induce expression of bacterial virulence pathways (17). These changes increase inflammation, which further favors the CDI disease state (25) through toxin-dependent and toxin-independent pathways. This leads to osmotic changes in the colon due to loss of colonocyte barrier function, making mucosal adherence more difficult for many bacteria and reducing a large proportion of the remaining and diminished bacterial population to a planktonic state, with their resources diverted toward functions such as osmotic regulation and run-and-tumble locomotion and away from population recovery. Concurrent with all of this is the enrichment of pathways for xenobiotic compound production, from bacterial and possibly fungal sources, potentially reinforcing a dysbiosis favoring CDI. Although Fig. S6 in the supplemental material describes the pathways with the strongest enrichment, it does not provide an exhaustive description of the pathogenesis of CDI, including the influence of the host inflammasome on this disease. As part of our team’s ongoing evaluation of these data, these pathways are currently a focus of animal research by our group to define their role in CDI.

This is the first tiered study of CDI using a combination of amplicon sequencing and matched metagenomics and metatranscriptomics. A smaller sample size is a limitation with this study, one reflecting cost constraints with metagenomics and metatranscriptomics. Our stringent inclusion criteria and the absence of differences in antibiotic exposure between those with and without CDI help to strengthen our conclusions despite this limitation. There is evidence that fungi are enriched in CDI, suggesting that there is a transkingdom interaction between fungi and bacteria in this disease; if this is demonstrated in further studies, it would introduce the concept of a fungus-associated bacteriome in CDI. A potential role for E. coli and *Pseudomonas* in CDI is also provided in this study, especially in terms of biofilm production, and the roles of host stress response as well as inflammation are further described as gut-related factors leading to the creation of niches potentially favoring C. difficile. In concert, these data describe mechanisms by which the causal dysbiosis for CDI may resist reversal, providing a possible, new explanation of CDI treatment failures. Future studies by the authors will investigate the roles of E. coli and *Pseudomonas* in CDI using animal models, as well as investigating whether the addition of antifungal therapy to C. difficile-directed antibiotics improves treatment success.

## MATERIALS AND METHODS

The Institutional Review Board of The Pennsylvania State Milton S. Hershey Medical Center approved this study. Each patient consented using an IRB-approved consent form prior to the collection of their stool sample.

### Patients.

A total of 49 inpatients admitted to the first author’s institution at the time of sample accrual for the treatment of various medical ailments were enrolled in this study between March 2017 and June 2017. Patients who were at least 18 years of age were eligible, with no upper age limit. As with prior studies from our group (14), patients receiving chemotherapy within 60 days of enrollment, those with inflammatory bowel disease, those with a history of a positive C. difficile test within 60 days of potential enrollment, those empirically started on C. difficile-directed antibiotics prior to stool testing, and those within 30 days of a mechanical bowel preparation were ineligible for inclusion.

Also similar to previous studies from our group (19), only diarrheal stools were collected for analysis in this study. As part of their routine clinical care, each patient with a clinical suspicion for CDI had a stool sample sent by their treating physician to PSHMC Clinical Microbiology for C. difficile testing using a commercially available nucleic acid amplification test designed to detect a highly conserved sequence within the *tcdA* gene. C. difficile positive and negative stool samples were preserved, after clinical testing, in a –80°C freezer until patients consented for inclusion in this study.

Table 6 provides a description of the number of stools subjected to amplicon sequencing (16S and ITS rRNA gene amplicon sequencing), as well as shotgun metagenomics and metatranscriptomics sequencing (described in the supplemental material). Briefly, fecal DNA extracts were subject to 16S rRNA gene and ITS2 Illumina tag PCR, pooled in equimolar ratios, gel purified, and sequenced on the Illumina MiSeq (16S rRNA libraries) and NextSeq (ITS libraries) platforms. Bacterial (16S rRNA gene) and fungal (ITS) sequences were quality filtered, clustered into operational taxonomic units (OTUs), and normalized using both the USEARCH and QIIME pipelines. Alpha and beta diversity calculations, as well as multivariate statistics, were performed as described in the supplemental material (Text S1). Fecal DNA was subjected to metagenomics library preparation using the Illumina Nextera XT kit. RNA extracted from fecal samples was converted to double-stranded cDNA, and libraries were prepared using the NuGEN Ovation kit. Samples that yielded detectable concentrations of high-integrity RNA and DNA were selected to maximize the number of matched metagenome/metatranscriptome samples for this study. Metagenomics and metatranscriptomics libraries were quantified, pooled, purified, and sequenced on the Illumina Hiseq4000 platform.

**TABLE 6:** Sample distribution of processed CDI^+^/CDI^−^ fecal samples

Sequencing technique	No. C. difficile positive	No. C. difficile negative	Total no.
16S rRNA	18	31	49
ITS	15	23	38
Metagenomics (MG)	12	14	26
Metatranscriptomics (MT)	7	14	21
Matched MG/MT	7	13	20

### 16S rRNA gene data processing.

Forward and reverse reads were merged using USEARCH7 (26) with a minimum overlap set to 200 bp. Using USEARCH7, paired sequences were quality filtered at a maximum expected error of 0.5% and were subsequently truncated at a length of 249 bp. Filtered reads maintained an average Phred Q score of 40.5 postfiltering. OTUs were picked *de novo* using the UPARSE algorithm (26) at 97% similarity. Taxonomy was assigned using UCLUST within QIIME 1.9.1 (27) using the Greengenes 16S rRNA gene database (13-5 release, 97%) (28). Results were compiled into a biological observation matrix (biom) format OTU table with a total count of 3,768,584 after singleton removal. All 49 samples produced the minimum number of quality-filtered sequences (>5,000 sequences per sample) for downstream analysis. A range of 12,010 to 178,904 sequences per sample was observed. Alpha diversity rarefaction curves were created within the QIIME 1.9.1 package (27) using an unrarified OTU table. Multiple rarefactions were performed on the 16S rRNA OTU table from all samples using a minimum depth of 100 sequences to a maximum depth of 12,010 sequences, with a step size of 794 for 20 iterations. Rarefactions then were collated and compared between disease states considering observed species, Chao1, PD whole-tree, and Heip’s evenness diversity metrics. Alpha diversity comparisons were conducted using a two-sample *t* test and nonparametric Monte Carlo permutations (*n *=* *999) within QIIME-1.9.1. 16S rRNA OTU tables were normalized using metagenomeSeq’s cumulative sum scaling (CSS) algorithm (29) for beta diversity and biomarker analysis. Beta diversity analyses were performed using a weighted UniFrac distance matrix and visualized within a three-dimensional principal coordinate analysis (PCoA) plot in EMPeror (29, 30). Analysis of similarity (ANOSIM) tests for significance were calculated within QIIME 1.9.1 to determine significance of clustering between disease cohorts.

Bipartite cooccurrence networks of bacterial and fungal taxonomy within C. difficile-positive (CDI^+^) and -negative (CDI^−^) samples were constructed as described in Lamendella et al. (19) with the following changes in protocol. For a taxon node to be included in the network, it needed to occur in at least 50% of the samples and have a Spearman’s rho threshold of at least 0.90. Spearman’s correlations were paired with two dissimilarity measures, Bray-Curtis and Kullback-Leibler, to calculate the distances between nodes. An edge selection of 75 was utilized for all generated networks. All three statistical measures ensured minimal significant correlations due to outliers or error in data composition. To adjust for multiple testing corrections, the Benjamini-Hochberg correction was used to adjust *P* values in the last network processing step. Bacterial and fungal taxa were labeled down to the lowest identified taxonomic ranking. Taxonomic nodes were sized by relative abundance and colored by phylum.

### ITS data processing.

Single-end ITS sequences were quality filtered at an expected error of less than 0.5% using USEARCH v7 (26). After quality filtering, reads were analyzed using the QIIME 1.9.1 software package (16). Of the 49 processed ITS libraries, 38 samples yielded at least 5,000 sequences for downstream processing. A total of 17,789,158 sequences were obtained after quality filtering and chimera analysis. Open-reference OTUs were picked and chimeras were removed using the UPARSE algorithm within USEARCH at 97% identity, and taxonomy assignment was performed against the UNITE database using BLAST within QIIME-1.9.1. OTUs with taxonomy assigned were organized into a BIOM-formatted OTU table, which was then summarized within QIIME 1.9.1. Alpha diversity analyses were conducted within the QIIME 1.9.1 sequence analysis package using an unrarified OTU table. Multiple rarefactions were conducted on sequences across all samples to a maximum rarefaction depth of 11,990 sequences, with 20 iterations at each step with a step size of 790. Alpha diversity was then collated and compared between disease states considering observed species richness, Chao1, and Heip’s evenness metrics. PCoA plots and ANOSIM tests for significance were generated from a weighted Jaccard distance matrix made within QIIME 1.9.1 from a CSS-normalized OTU table (29).

### Metagenomic and metatranscriptomic taxonomic/functional gene profiling and comparative analysis.

Taxonomic classification and bacterial species relative abundances were calculated with MetaPhlAn2 using default settings (31). Unassigned taxa were discarded for downstream taxonomic comparisons, and species consisting of less than 0.01% of identified marker sequences across all samples were discarded to reduce data noise. Taxonomic output from all metagenomes and metatranscriptomes were merged into two respective *.tsv* tables for downstream analysis of each data set. Functional gene annotation and quantification of filtered sequence data were conducted using HUMAnN2 (http://huttenhower.sph.harvard.edu/humann2; v.0.9.9) against the Uniref90 functional gene database (32). Default HUMAnN2 settings were utilized for functional gene annotation. Generated reads per kilobase (RPK) counts of Uniref90 annotations were regrouped as KEGG orthologies (KO) and underwent CPM normalization to account for differences in sequencing depth between samples.

CPM-normalized KO counts were grouped into KEGG pathways within HUMAnN2 using a custom python script for LDA effect size (LEfSe) plotting. For each respective metagenome and metatranscriptome data set, relative abundances of taxonomic profiles and functional pathways were multiplied by 1 million and formatted as described in Segata et al. (33). Comparisons were made with “disease” as the main categorical variable (“class”). Alpha levels of 0.05 were used for both the Kruskal-Wallis and pairwise Wilcoxon tests. LDA scores greater than 2.0 are displayed for taxonomy and functional (KEGG) pathways. Resulting taxonomic and functional gene pathway biomarkers between CDI^−^ and CDI^+^ individuals were then plotted in cladogram structures for the metagenome and metatranscriptome data sets, respectively. CPM-normalized KEGG orthology pathway tables were stratified by bacterial taxa within HUMAnN2 and were subsequently summarized by bacterial taxa using a suite of custom python scripts and were imported into an R environment. The R packages ggplot2 (34) and plotly (35) were then utilized to produce visualizations displaying the eight most abundant bacterial contributors, based on the total CPM normalized gene count, to each pathway.

Partial least-squares discriminant analysis (PLS-DA) and complementary receiver operating characteristic (ROC) curve plotting was performed considering CPM-normalized KO metagenome and metatranscriptome data using the mixOmics R package (36). The PLS-DA model was trained using a 10-fold cross validation, and this model underwent 150 iterations. Metatranscriptome CPM-normalized KO counts were averaged within CDI^+^ and CDI^−^ cohorts, respectively, for Pathview (version 3.6) plotting (37). Average CPM-normalized expression counts within CDI^+^/CDI^−^ cohorts were log +1 transformed and were plotted against the flagellar assembly (KEGG map02040), biofilm formation-E. coli (KEGG map02026), two-component system (KEGG map02020), quorum-sensing (KEGG map02024), and linoleic acid metabolism (KEGG map00591) functional pathway maps within the Pathview R package.

Multivariate association with linear models (MaAsLin) was conducted to find associations between clinical metadata of interest, including CDI status as well as antibiotic treatment status and identified metatranscriptome functional pathways. Functional pathway expression data underwent relative abundance normalization prior to MaAsLin analysis as required by the protocol (38). Enrichment results were displayed at a false discovery rate (FDR)-corrected *P* value (*q* value) of <0.40.

Additional sample preparation and bioinformatics methodologies are detailed in the supplemental material.

### Data availability.

For all sequencing results, an archive is publicly available at NCBI BioProject ID PRJNA478949 under the SRA accession number SRP151803.

10.1128/mSphere.00454-19.6 FIG S5 Significantly enriched (LDA > 2.0; *P < *0.05) KEGG functional gene pathways within CDI^+^ (red) and CDI^−^ (green) cohorts were visualized in cladogram structures considering the metagenomic dataset. Two-component system, phosphotransferase system, flagellar assembly, and biofilm formation-Vibrio cholerae were identified as enriched pathways within CDI^+^ individuals, whereas histidine metabolism, aminoacyl tRNA biosynthesis, N-glycan biosynthesis, glycosphingolipid biosynthesis-ganglio series, and valine, leucine, and isoleucine biosynthesis were observed among the enriched biomarker pathways of CDI^−^ individuals. Download FIG S5, TIF file, 0.3 MB. Copyright © 2019 Stewart et al. 2019 Stewart et al. This content is distributed under the terms of the Creative Commons Attribution 4.0 International license.

10.1128/mSphere.00454-19.7 FIG S6 Significantly enriched (LDA > 2.0; *P < *0.05) KEGG functional gene pathways within CDI^+^ (red) and CDI^−^ (green) cohorts were visualized in cladogram structures considering the metatranscriptomic dataset. Two-component system, quorum sensing, flagellar assembly, biofilm formation-Pseudomonas aeruginosa, linoleic acid metabolism, and alpha-linoleic acid metabolism were identified as enriched pathways within CDI^+^ individuals, whereas carbon fixation pathways and methane metabolism were among the enriched biomarker pathways of CDI^−^ individuals. Download FIG S6, TIF file, 0.3 MB. Copyright © 2019 Stewart et al. 2019 Stewart et al. This content is distributed under the terms of the Creative Commons Attribution 4.0 International license.

10.1128/mSphere.00454-19.8 FIG S7 Functional gene pathway contribution plot displays CPM-normalized expression counts of prominent functional gene pathways mapped to the *Clostridium* within CDI^−^ (green) and CDI^+^ (red) individuals. Here, the top 20 pathways mapped to *Clostridium* with the highest CPM-normalized count across all samples are plotted on the *y* axis, and the percentage of expression within CDI^−^ and CDI^+^ individuals is plotted on the *x* axis. An increase in *Clostridium* expression of genes related to two-component systems (4.76-fold increase in CDI^+^ individuals) and bacterial secretion systems (2.27-fold increase in CDI^+^ individuals) were more highly expressed within CDI^+^ individuals. Download FIG S7, TIF file, 0.3 MB. Copyright © 2019 Stewart et al. 2019 Stewart et al. This content is distributed under the terms of the Creative Commons Attribution 4.0 International license.

10.1128/mSphere.00454-19.1 TABLE S1 MaAsLin antibiotic treatment status enrichment results. Download Table S1, DOCX file, 0.01 MB. Copyright © 2019 Stewart et al. 2019 Stewart et al. This content is distributed under the terms of the Creative Commons Attribution 4.0 International license.

10.1128/mSphere.00454-19.1 TEXT S1 Supplemental materials and methods. Download Text S1, DOCX file, 0.02 MB. Copyright © 2019 Stewart et al. 2019 Stewart et al. This content is distributed under the terms of the Creative Commons Attribution 4.0 International license.

10.1128/mSphere.00454-19.1 TEXT S2 Supplemental results. Download Text S2, DOCX file, 0.02 MB. Copyright © 2019 Stewart et al. 2019 Stewart et al. This content is distributed under the terms of the Creative Commons Attribution 4.0 International license.
