# Supplementary material for: Integrated Meta-omics Reveals a Fungus-Associated Bacteriome and Distinct Functional Pathways in Clostridioides difficile Infection
Source: mSphere. 2019 Aug 28;4(4):e00454-19. doi: 10.1128/mSphere.00454-19 (PMC6714892; doi:10.1128/mSphere.00454-19)
Supplement: TABLE S1 [file mSphere.00454-19-st001.docx]

| **TABLE S1** MaAsLin antibiotic treatment status enrichment results | | | |
| --- | --- | --- | --- |
| **Pathway** | **Coefficient (>0 = Antibiotics+, <0 = Antibiotics-)** | ***P-*value** | **Q-value** |
| Arginine and proline metabolism | 0.01488 | 0.0032 | 0.0895 |
| Isoquinoline alkaloid biosynthesis | 0.01715 | 0.0065 | 0.1430 |
| Pentose and glucuronate interconversions | 0.02493 | 0.0096 | 0.1649 |
| Tropane piperidine and pyridine alkaloid biosynthesis | 0.01757 | 0.0126 | 0.1979 |
| Sulfur metabolism | 0.02376 | 0.0147 | 0.2103 |
| Autophagy yeast | 0.00295 | 0.0154 | 0.2121 |
| HIF signaling pathway | -0.01107 | 0.0163 | 0.2151 |
| **Biofilm formation Escherichia coli** | **0.01873** | **0.0179** | **0.2270** |
| Tyrosine metabolism | 0.02067 | 0.0192 | 0.2341 |
| Caprolactam degradation | -0.00516 | 0.0216 | 0.2547 |
| Pantothenate and CoA biosynthesis | -0.00813 | 0.0247 | 0.2552 |
| **Flagellar assembly** | **0.02637** | **0.0288** | **0.2791** |
| Phosphatidylinositol signaling system | 0.01393 | 0.0362 | 0.3232 |
| **Biofilm formation Vibrio cholerae** | **0.02563** | **0.0396** | **0.3410** |
| Flavone and flavonol biosynthesis | 0.00546 | 0.0403 | 0.3410 |
| Galactose metabolism | -0.01723 | 0.0429 | 0.3450 |
| Ribosome | -0.04598 | 0.0459 | 0.3605 |
| Glycolysis Gluconeogenesis | -0.00627 | 0.0478 | 0.3652 |
| **Lipopolysaccharide biosynthesis** | **0.02963** | **0.0487** | **0.3652** |
| Steroid hormone biosynthesis | 0.01415 | 0.0587 | 0.3916 |
| Phenylalanine tyrosine and tryptophan biosynthesis | 0.01513 | 0.0593 | 0.3916 |
| Phosphonate and phosphinate metabolism | 0.02208 | 0.0609 | 0.3939 |
