# Supplementary material for: Integrated Meta-omics Reveals a Fungus-Associated Bacteriome and Distinct Functional Pathways in Clostridioides difficile Infection
Source: mSphere. 2019 Aug 28;4(4):e00454-19. doi: 10.1128/mSphere.00454-19 (PMC6714892; doi:10.1128/mSphere.00454-19)
Supplement: TEXT S1 [file mSphere.00454-19-s0001.docx]

**SUPPLEMENTAL MATERIALS AND METHODS**

**16S and ITS rRNA gene amplicon processing and analysis**

**DNA extraction.** Fecal samples (N = 49) were shipped to Juniata College on dry ice for microbiome library preparation and analysis. A Qiagen DNeasy Powersoil DNA Isolation kit was used following the manufacturer protocol on approximately 0.25g of each fecal sample (Qiagen, Frederick, MD, USA). DNA concentrations were then quantified with the Qubit 2.0 Fluorometer High Sensitivity dsDNA kit (Life Technologies, Carlsbad, CA, USA) according to the manufacturer’s instructions. For each round of nucleic acid (N = 2) extraction, negative control blanks were run in parallel with fecal samples, using 500 µL of ultrapure DNA/RNA free water (Ambion, Inc. Thermo Fisher Scientific). All extraction blanks were run through 16S and ITS Illumina-tag PCR and did not produce any visible PCR product. A total of 10% of all PCR reactions were set up as negative controls, with ultra pure DNA/RNA free water (Ambion, Inc. Thermo Fisher Scientific) used in place of template DNA. No negative control PCR reactions produced visible PCR products.

**16S/ITS rRNA gene PCR amplification.** Ilumina itag (Illumina, San Diego, CA, USA) polymerase chain reactions were performed using the Earth Microbiome Project’s 16S Protocol for 16S rRNA gene amplicon sequencing. A total volume of 25 μL was used for each reaction, these contained 0.2 μM non-Barcoded Primer, 1X Buffer mixture, 0.8 μM dNTP mixture, 0.625U Ex Taq DNA Polymerase (Takara Bio USA, Mountain View, CA, USA), 1M Betaine, 2 μL template DNA and enough PCR Grade water to bring the reaction to 25 μL. Amplification was carried out on a Bio-Rad T100 thermal cycler using the following cycling conditions: 94°C for 3 minutes to denature DNA, with 35 cycles at 94°C for 45 seconds, 50°C for 60 seconds, and 72°C for 90 seconds. Final extension was at 72°C for 10 minutes with subsequent holding at 4°C until further processing.

For ITS processing, the volume of each reaction was 25 μL and contained (final concentrations) 1X PCR buffer, 0.8 mM dNTP's, 0.625 U Ex Taq DNA Polymerase (Takara), 0.2 μM ITS1F forward primer, 0.2 μM ITS2R reverse barcoded primer and 2 µL of template DNA diluted to between 1:5 and 1:100 DNA:sterile water to optimize the reaction due to high extract concentrations. Amplification was carried out on a T100 Thermal Cycler (Bio-Rad, Hercules, CA, USA) using the following cycling conditions: 94°C for 1 minute, followed by 35 cycles of 94 °C for 30 seconds, 52°C for 30 seconds, and 68°C for 30 seconds, with final extension at 68 °C for 7 min followed by holding at 4°C until further processing. A total of 10% of all PCR reactions were set up as negative controls, with ultra pure DNA/RNA free water (Ambion, Inc. Thermo Fisher Scientific) used in place of template DNA. No negative control PCR reactions produced visible PCR products. A total of 49 samples produced amplified PCR product for 16S and ITS rRNA gene targets, respectively.

**Library purification and verification methods.** PCR products were combined (pooled) in equimolar amounts. The pooled PCR products were then run on a 2% agarose gel with GelStar Nucleic Acid Gel Stain (Lonza, Walkersville, MD, USA) for visualization. Bands of expected product length were cut from the gel using sterile scalpels and were subsequently purified using the QIAquick Gel Purification Kit (Qiagen). The pure library was then quantified using the Qubit 2.0 Fluorometer double stranded DNA high sensitivity assay (Life Technologies). Finally, each library on the sequencing run was combined (multiplexed) into one sequencing library by normalizing each library’s input based on the number of samples per sequencing run to ensure even sequencing and coverage.

Prior to submission for sequencing, libraries were quality checked using a 2100 Bioanalyzer high sensitivity DNA analysis kit (Agilent Technologies, Santa Clara, CA, USA). Purified 16S rRNA gene libraries were size verified using the Fragment Analyzer on the ABI3730 and were quantified with a KAPA Library quantification kit (Kapa Biosystem, Wilmington, MA, USA). After dilution with EBT (Illumina) to a final concentration of 2 nM containing 15% PhiX V3 library control (Illumina), the library pools were denatured for 5 minutes in an equal volume of 0.2M NaOH, then further diluted to 8 pM in HT1 buffer (Illumina) and were sequenced using an Illumina MiSeq V2 500 cycle kit cassette with 16S rRNA library sequencing primers set for 250 basepair, paired-end reads. Overall sequencing run performance was evaluated by determining whether the sequencing runs met the Illumina specifications for quality scores and data output. Actual run performance varied based on sample type, quality, and clusters passing quality filtering measures. Specifications were based on the Illumina PhiX control library at supported cluster densities. Purified ITS libraries were pooled and sequenced on the Illumina NextSeq platform using the 150 bp single end chemistry at University of California Davis Genome Center.

**Metagenome and metatranscriptome processing and sequencing.** For metagenome and metatranscriptome library preparation, a subset of samples that produced the highest concentrations of high integrity RNA and ample DNA were selected to maximize the number of matched metagenome/metatranscriptome samples for this study. A minimum concentration of 5 ng/uL of RNA with a RIN >6 was used for sample selection. A total of 32 metagenomes and 28 metatranscriptomes produced libraries that yielded sufficient concentration and quality for sequencing. Sufficient concentration was determined as a library exceeding 0.8 nM for a single library which was subsequently pooled in equimolar ratios with the other samples the same sequencing run.

**Metagenome library preparation.** DNA extractions were conducted as described in our Supplemental material. A total of 1 ng of DNA per sample underwent Nextera XT (Illumina) tagmentation and library preparation. Quality of final libraries was assessed using the High Sensitvity DNA assay on the Agilent 2100 Bioanalyzer (Agilent). Equimolar amounts of library were pooled and purified using a QIAquick gel purification kit, in which a library sized 250-450 bp was selected for and purified (Qiagen).

**Metatranscriptome RNA extraction and library preparation.** For RNA extraction, approximately 0.25 grams of each fecal sample underwent RNA extraction using the RNeasy PowerMicrobiome Kit (Qiagen). The RNeasy PowerMicrobiome kit utilizes a cell lysis protocol that is similar to the PowerSoil DNA isolation kit, which relies on the addition of each fecal sample to a beaded tube in combination with Lysis buffer (Solution PM1/β- mercaptoethanol) and are subsequently vortexed within the Disruptor Genie for 10 minutes. All RNA extracts were quantified using the Qubit DNA High Sensitivity Kit (Invitrogen, Carlsbad, CA, USA) to confirm complete DNase treatment of the RNA extracts (DNA concentration <0.05 ng/uL). Subsequently, approximately 100 ng of extracted RNA was subject to NuGEN Ovation (NuGEN Technologies, San Carlos, CA, USA) double stranded cDNA synthesis and metatranscriptome library preparation. Five samples with lower RNA yields underwent preparation with less than 100 ng of input RNA (26-68 ng total RNA). Quality of the final library was assessed using a high sensitivity bioanalyzer chip (Agilent). The same library quantification, pooling, and purification methods were used as described above. Purified metagenome and metatranscriptome libraries underwent sequencing on the Illumina HiSeq4000 following a 2 x 150bp index run at the UC Davis Genome Center.

**Meta-‘omics’ bioinformatic analysis**

***Quality filtering of raw data***

Raw read quality was assessed using the program FastQC to obtain average Q scores across the read length of all R1 and R2 fastq files (1). The program Trimmomatic (version 0.36) was used to quality filter and pair the raw sequence data (2). A sliding window filtration was utilized to cut reads at a four-base average Q score of ≤28; reads trimmed below 100 bp were discarded. Paired R1 and R2 files were concatenated and evaluated with the KneadData (version 0.5.4 - https://bitbucket.org/biobakery/kneaddata) pipeline using default settings to remove human host DNA reads from filtered sequence data. 26 and 21 samples produced the minimum number of quality filtered sequences (N = 800,000) for downstream metagenomics and metatranscriptomics analyses, respectively.

**REFERENCES**

1. Andrews S. FastQC: a quality control tool for high throughput sequence data. 2010; <http://www.bioinformatics.babraham.ac.uk/projects/fastqc>. Accessed February 10, 2018.

2. Bolger AM, Lohse M, Usadel B. Trimmomatic: a flexible trimmer for Illumina sequence data. Bioinformatics. 2014 Aug 1;30(15):2114-20. doi: 10.1093/bioinformatics/btu170.
