# Supplementary material for: Integrated Meta-omics Reveals a Fungus-Associated Bacteriome and Distinct Functional Pathways in Clostridioides difficile Infection
Source: mSphere. 2019 Aug 28;4(4):e00454-19. doi: 10.1128/mSphere.00454-19 (PMC6714892; doi:10.1128/mSphere.00454-19)
Supplement: TEXT S2 [file mSphere.00454-19-s0002.docx]

**SUPPLEMENTAL RESULTS**

**Bacterial and fungal community profiling (16S rRNA/ITS gene) results**

**Sequencing results.** Of the 49 collected fecal samples, a total of 49 and 39 samples yielded enough high quality sequence data (>5,000 filtered sequences per sample) for 16S and ITS rRNA gene amplicon data analysis, respectively. A total of 1,244 bacterial OTUs and 2,239 fungal OTUs were observed. Considering the fungal ITS dataset, the *candida* were identified as the most abundant fungal genus, and yielded an increased average relative abundance within CDI- individuals (76.7%) in comparison to the CDI+ (54.9%) cohort. Within the bacterial 16S rRNA dataset, an increase in the average relative abundance of Enterobacteriaceae sp. within CDI+ (15.1%) individuals in comparison to CDI- (5.3%) samples. Additionally, Clostridiales sp. were elevated within CDI+ individuals (4.6%, CDI- = 1.1%). Within CDI- samples, an increase in relative abundance of commensal gut bacteria including the *lactobacillus* (CDI- = 4.0%, CDI+ = 0.3%) and *ruminococcus* (CDI- = 1.6%, CDI+ = 0.6%) were identified as more abundant. Prominent bacterial and fungal genera were summarized within each sample and are provided in (Additional file 2: Fig. S1).

**Metagenomic and metatranscriptomic results.** Of the 49 collected fecal samples, a subset of 32 samples were selected and processed for metagenome and metatranscriptome library preparation. A total of 32 metagenome libraries and 27 metatranscriptome libraries bypassed quality control for Illumina HiSeq sequencing. After quality filtration, 26 metagenome and 21 metatranscriptome samples yielded enough high quality sequence data (>400,000 sequences per sample) for HUMAnN2 annotation. Filtered metagenome samples yielded a range of 617,000 - 8,092,292 paired sequences (median count: 2,586,456 sequences), whereas filtered metatranscriptome samples yielded a range of 413,928 - 64,958,250 sequences (median count: 5,105,588 sequences). Across all metagenome samples, a total of 428,026 Uniref90 annotations were regrouped as 6,912 KEGG orthologies for downstream bioinformatics analysis. Considering the metatranscriptome data a total of 263,292 Uniref90 annotations were regrouped as 4,394 KEGG orthologies.
